# Impact of *ABCC2* 1249G>A and −24C>T Polymorphisms on Lacosamide Efficacy and Plasma Concentrations in Uygur Pediatric Patients With Epilepsy in China

**DOI:** 10.1097/FTD.0000000000001003

**Published:** 2023-10-08

**Authors:** Ting Zhao, Hong-jian Li, Hui-lan Zhang, Jie Feng, Jing Yu, Ting-ting Wang, Yan Sun, Lu-hai Yu

**Affiliations:** * Department of Pharmacy, People's Hospital of Xinjiang Uygur Autonomous Region;; † Institute of Clinical Pharmacy of Xinjiang Uygur Autonomous Region, People's Hospital of Xinjiang Uygur Autonomous Region; and; ‡ Department of Neurology, Xinjiang Hospital of Beijing Children's Hospital, Children's Hospital of Xinjiang Uygur Autonomous Region, Urumqi, China.

**Keywords:** *ABCC2*, epilepsy, efficacy, lacosamide, plasma concentrations

## Abstract

We aimed to evaluate the effect of the *ABCC2* 1249G>A (rs2273697) and −24C>T (rs717620) polymorphisms on lacosamide (LCM) plasma concentrations and the efficacy of LCM in Uygur pediatric patients with epilepsy.

**Methods:**

We analyzed 231 pediatric patients with epilepsy, among which 166 were considered to be LCM responsive. For drug assays, 2–3 mL of venous blood was collected from each patient just before the morning LCM dose was administered (approximately 12 hours after the evening dose, steady-state LCM concentrations). The remaining samples after routine therapeutic drug monitoring were used for genotyping analysis. The χ^2^ test and Fisher exact test were utilized for comparative analysis of the allelic and genotypic distribution of *ABCC2* polymorphisms between the LCM-resistant and LCM-responsive groups. The Student *t* test or Mann–Whitney *U* test was conducted to analyze differences in plasma LCM concentration among pediatric patients with epilepsy with different genotypes.

**Results:**

Patients with the *ABCC2* 1249G>A GA genotype (0.7 ± 0.3 mcg/mL per kg/mg) and AA genotype (0.5 ± 0.3 mcg/mL per kg/mg) showed significantly (*P* < 0.001) lower LCM concentration-to-dose (CD) ratios than patients with the GG genotype (1.0 ± 0.4 mcg/mL per kg/mg). Moreover, patients with the *ABCC2* −24C>T CT genotype (0.6 ± 0.2 mcg/mL per kg/mg) and TT genotype (0.6 ± 0.3 mcg/mL per kg/mg) presented a significantly (*P* < 0.001) lower LCM CD ratio than patients with the CC genotype (1.1 ± 0.4 mcg/mL per kg/mg).

**Conclusions:**

The *ABCC2* 1249G>A (rs2273697) and *ABCC2* −24C>T (rs717620) polymorphisms can affect plasma LCM concentrations and treatment efficacy among a population of Uygur pediatric patients with epilepsy, causing these patients to become resistant to LCM. In clinical practice, ABCC2 polymorphisms should be identified before LCM treatment, and then, the dosage should be adjusted for pediatric patients with epilepsy accordingly.

## BACKGROUND

Lacosamide (LCM) is a novel antiseizure medication that plays a unique anticonvulsant role by selectively inhibiting the activation of voltage-gated sodium channels.^1^ In August and October 2008, LCM was granted approval in Europe and the United States, respectively, for the treatment of partial-onset seizures with or without secondary generalization in adults, adolescents, and children age 4 years with epilepsy.^2,3^ Multiple studies have shown that LCM is associated with favorable short-term and long-term efficacy, tolerability, and safety in the treatment of epileptic patients.^4–6^ LCM was granted approval in China in 2018. A case report of 72 children with focal epilepsy (the mean age was 7.2 years; range, 0.9–14 years) undergoing treatment at the People's Hospital of Xinjiang Uygur Autonomous Region in Uygur, China, has been published; 50 (69%) of the children responded to LCM therapy, which was associated with a reduction of more than 50% in the frequency of seizures.^7^ However, our routine drug monitoring studies showed an increase in the number of children with epilepsy who developed drug resistance. Resistance is defined as failure of treatment with LCM as monotherapy or in combination with other antiseizure medications for at least 12 months at the maximal tolerated doses, with persistence of epileptic seizures.

Multidrug transporters play a major role in the mechanism of drug resistance. Abnormally expressed multidrug transporters are known to prevent antiseizure medications (ASMs) from entering the brain tissue, thereby reducing the concentration of ASMs at specific targets in the brain and resulting in resistance to epilepsy drugs.^8^ P-glycoprotein (P-gp) is the first and most widely studied multidrug transporter.^9^ P-gp can pump ASMs into blood by hydrolyzing energy provided by ATP; it reduces drug concentration at the epileptic foci, thereby inhibiting ASMs in the epileptic discharge of nerve cells.^10^ P-gp is encoded by the multidrug resistance gene.^11^ Mutations in the multidrug resistance gene that encodes P-gp can affect the efflux of endothelial cell transporters in the blood–brain barrier. This, in turn, will affect the pharmacokinetic parameters and blood drug concentrations of ASMs, further impairing their efficacy and toxicity as well as inducing drug resistance.^12^

*ABCC2* (also known as multidrug resistance protein 2, MRP2) is an ATP-binding cassette transporter that is largely responsible for the active efflux of many drugs. Single nucleotide polymorphisms (SNPs) of *ABCC2* can influence the expression and function of the resultant proteins and are likely associated with drug resistance among patients with epilepsy.^13^ The most studied SNPs in this gene include 1249G>A (rs2273697) and −24C>T (rs717620).^14^ Grewal et al^15^ revealed that altered functionality of ABCB1 and *ABCC2* can affect the disposition and bioavailability of ASMs, thereby interfering with antiseizure medication therapy. Xue et al suggested that the *ABCC2* rs717620 polymorphism is associated with resistance to ASMs among Uygur patients with epilepsy.^16^ In addition, Chen et al^17^ discovered that in a subgroup of generalized seizure, *ABCC2* rs3740066 CC carriers had a higher frequency of valproic acid resistance than TC + TT carriers (*P* < 0.05).

However, there has been no comprehensive investigation into the relationship between SNPs in *ABCC2* with plasma LCM concentration. Therefore, this study was conducted to evaluate the association of the 1249G>A and −24C>T genotypes of *ABCC2* as well as their haplotypic and diplotypic combinations with the plasma concentration and efficacy of LCM in Uygur pediatric patients with epilepsy. The purpose of this study was to provide a valuable tool for predicting the clinical efficacy of LCM before treatment and to contribute to the individual use of ASMs among Uygur pediatric patients with epilepsy.

## MATERIALS AND METHODS

### Subjects

A total of 296 pediatric patients with epilepsy who received LCM treatment at the People's Hospital of Xinjiang Uygur Autonomous Region, China, in 2018–2021 were included in this study. Sixty-five pediatric patients with epilepsy were excluded owing to incomplete data. Overall, 231 pediatric patients with epilepsy who underwent LCM treatment were included.

All pediatric patients met the criteria for a diagnosis of epilepsy, as issued by the International League against Epilepsy in 2017.^18^ This study was granted approval by the Ethics Committee of People's Hospital of Xinjiang Uygur Autonomous Region, Xinjiang, China (Ethical Approval Number: KY2019120614). The parents of all patients signed an informed consent form.

All subjects were regularly treated with LCM tablets, in accordance with the study protocol. Seizure frequencies at 3, 6, and 12 months after the initiation of LCM therapy were recorded and compared with the baseline monthly frequency. Participants were presumed to be responsive if treatment with LCM at the maximum tolerated dose for at least 3 months, either as monotherapy or in combination with other ASMs, led to a reduction in the frequency of seizures by ≥50%.^19^ In addition, participants were presumed to be resistant if treatment with LCM at the maximum tolerated doses for at least 12 months, either as monotherapy or combined with other ASMs, failed to reduce the frequency of seizures by ≥50% and epileptic seizures persisted. Pediatric patients were divided into either the responsive or resistant group according to their seizure frequency.

### Therapeutic Drug Monitoring of LCM

All pediatric patients were administered LCM tablets 2 times a day. The initial dose of LCM was 2 mg/kg daily, and this dose was increased once every week. The target dose was 5–20 mg/kg daily for 3–4 weeks, and the target range of LCM serum concentrations was 2.5–15 mcg/mL. Once the maintenance dose was reached, the first blood sample was collected. If the LCM serum concentration did not reach the target range, blood sampling was repeated after a week of dose adjustment.

For drug concentrations assays, 2–3 mL of venous blood was obtained from each patient just before the morning LCM dose was administered (approximately 12 hours after the evening dose; trough concentration). This study used a single blood sample at a single point in time. Plasma LCM concentrations were measured in only 1 blood sample per patient at the last follow-up. EDTA anticoagulant tubes containing the blood samples were immediately centrifuged at ×4000*g* (−4°C) for 5 minutes, and the resulting plasma was transferred into a clean tube and stored at −80°C. The leftover samples after routine therapeutic drug monitoring were used for genotyping analysis.

The extraction process was initiated by adding 300 µL of organic deproteinization solution (Abbott Laboratories, Shanghai, China) to 100 µL of plasma sample. Next, the solution was vortexed for 2 minutes and then centrifuged at ×12,100*g* for 10 minutes. The upper organic layer was then collected and placed into a clean glass tube. Vials were placed within a thermostatic autosampler (10°C), and 0.5-µL aliquots were used for ultra-performance liquid chromatography (UHPLC) assay.

Plasma LCM concentrations were quantified using a validated UHPLC method.^20^ All LCM samples were evaluated at the individualized research laboratory of the Institute of Clinical Research of our hospital. All samples were analyzed in batches once a week on average. The chromatography instrument was Waters ACQUITY UPLC BEH (C18, 2.1 × 100 mm, 1.7 μm, Shanghai, China). The mobile phase was ammonium dihydrogen phosphate solution (10 mmol/L)—methanol (55:45, vol/vol; pH adjusted to 4.0 with phosphoric acid). The flow rate was 0.2 mL/min. The injection volume was 2 μL, and the detection wavelength was 210 nm. The peak area of LCM in serum samples was linear and within the concentration range of 0.5–40 mcg/mL (y = 0.0494CLCM +0.0222, r2 ≥ 0.9997). The lower limit of quantification (LLOQ) of LCM was determined to be 0.25 mcg/mL. After successive dilutions of the lowest calibration standard, the low quality control (LQC) of LCM was set to 0.5 mcg/mL. The intraday and interday precision values, as measured by relative SD values, were between 1.4% and 4.5%. The recovery ranged from 96.6% to 106.2%. All plasma samples were stable for up to 3 hours at ambient temperature (24 hours at 4°C and 30 days at −80°C) and after 6 successive freeze–thaw cycles (24 hours per cycle).

### Genotyping

The *ABCC2* polymorphisms of all pediatric patients were analyzed in the research laboratory of the Institute of Clinical Pharmacy of our hospital. All samples were analyzed in 3 batches by professionals with PCR work certificates. Genomic DNA was extracted from whole blood using a standard method (http://www.qiagen.com). Two SNPs in the *ABCC2* gene (1249G>A and −24C>T) were genotyped using a PCR assay using Big Dye (Applied Sanger Sequencing Technologies, Hangzhou, China), followed by restriction fragment length polymorphism (RFLP) analysis.

The PCR and RFLP products were analyzed by gel electrophoresis on a 2% agarose gel. PCR amplification of the polymorphism site was performed using the following forward and reverse primers: *ABCC2* rs2273697: 5′-GTAAGATTTGGAGGCAAGAAGTC-3′ and 5′- CCCAACACCTGCTAAGACTGAG-3′; *ABCC2* rs717620: 5′- CCAATTGCACATCTAACATTTCTG-3′ and 5′-TAACGATTAAATGGTTGGGATG-3′. Table 1 presents detailed information on the primers and the target fragment sizes.

**TABLE 1.** Sequences of the Primers Used in This Study and the Size of Amplicons for Each SNP

SNP	Forward Primer	Reverse Primer	Amplicon Size (Base Pair)
ABCC2 1249G>A (rs2273697)	GTAAGATTTGGAGGCAAGAAGTC	CCCAACACCTGCTAAGACTGAG	350
ABCC2 −24C>T (rs717620)	CCAATTGCACATCTAACATTTCTG	TAACGATTAAATGGTTGGGATG	302

**FIGURE 1.**
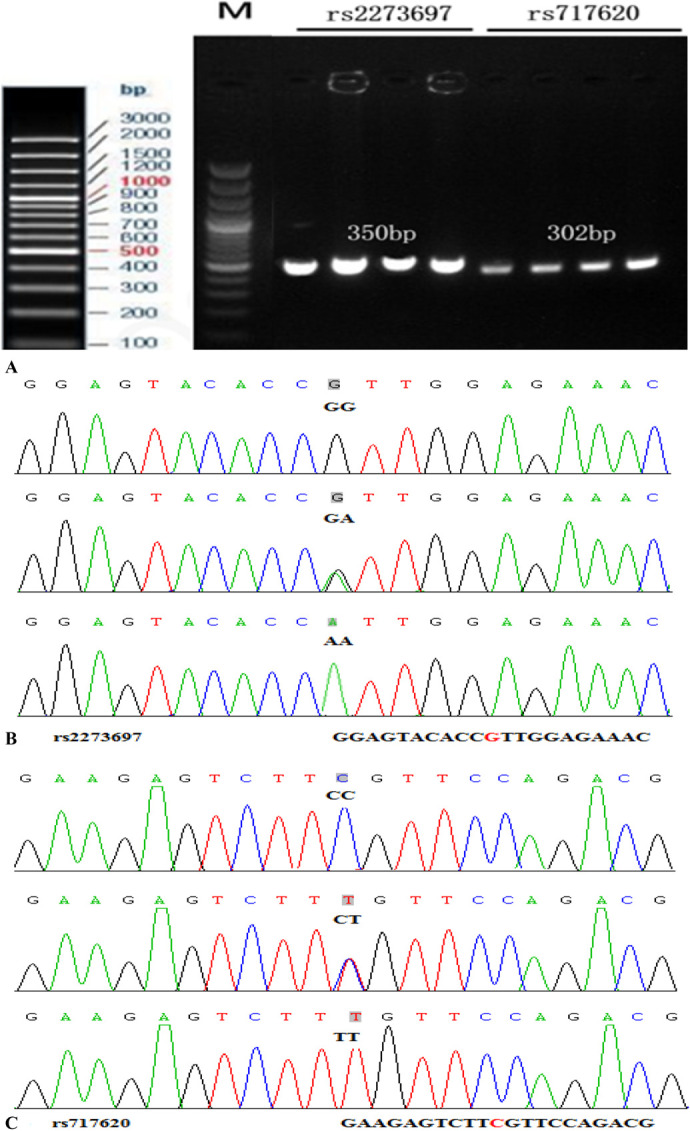
Determination of *ABCC2* rs2273697 and rs717620 genotypes by gel electrophoresis after PCR-RFLP, followed by validation through DNA sequencing. M represents marker. A, PCR amplifications of the *ABCC2* rs2273697 and rs717620 loci, digested by BanI. B, DNA sequence of the rs2273697 genotype. C, DNA sequence of the rs717620 genotype.

The PCR amplification was performed in a 25 mL reaction mixture consisting of 2.5 mL 10X PCR Buffer, 1 mL dNTP (10 μM), 1 mL forward primer (10 μM), 1 mL reverse primer (10 μM), 0.2 mL Taq enzyme (5 U/mL), 1 mL gDNA, and 25 mL ddH2O. The cycling conditions used to evaluate the 2 SNPs (rs2273697 and rs717620) were as follows: initial denaturation at 95°C for 5 minutes, followed by 30 cycles of denaturation, annealing, and extension at 94°C for 30 seconds, 63°C for 30 seconds, and 72°C for 60 seconds, respectively. A final extension step was performed at 72°C for 10 minutes Figure 1 presents the results of gel electrophoresis and DNA sequencing analysis for each genotype.

### Statistical Analysis

Statistical analyses were performed using SPSS (version 4.0.100.1124; Chicago, IL, Beijing, China). A *P* value of <0.05 was considered to be statistically significant. Linkage disequilibrium analysis and haplotype construction were conducted by the SHEsis online software^21^ to determine whether the research subjects were representative of the entire group. The χ^2^ (χ2) test was performed to compare allelic and genotypic distribution of *ABCC2* gene polymorphisms (1249G>A and −24C>T) between the drug-resistant and drug-responsive groups. The combined effects of SNPs and nongenetic factors were further assessed using a multivariate regression model. To evaluate differences in plasma LCM concentrations among pediatric patients with epilepsy with different genotypes, the Student *t* test or analysis of variance (ANOVA) was performed for comparisons of continuous data and the Mann–Whitney *U* test was used for comparisons of nonparametric continuous data.

## RESULTS

### Characteristics of Pediatric Patients

The clinical and demographic characteristics of all enrolled patients are summarized in Table 2. In total, 231 Uygur pediatric patients with epilepsy (aged 4–14 years) were included in this study. The mean age at the initiation of LCM therapy was 7.7 ± 4.0 years. Overall, 60% of the patients were male (n = 139). The LCM maintenance dose ranged from 25 to 400 mg/d. After normalization by weight and daily dose, the plasma LCM concentration was determined to be 0.9 ± 0.4 mcg/mL per mg/kg. At the last follow-up (at 3, 6, or 12 months after the initiation of LCM therapy), 52 pediatric patients were on LCM monotherapy, 113 patients received valproic acid combination therapy, 69 patients received levetiracetam combination therapy, 65 patients received oxcarbazepine combination therapy, 29 patients received lamotrigine combination therapy, 3 patients received topiramate combination therapy, and 2 patients received clonazepam combination therapy.

**TABLE 2.** Statistical Analysis of Qualitative and Quantitative Variables Between the Effective and Ineffective Groups (Mean ± Standard Deviations)

Characteristic	Total Group, n (%)	Responsive Group (n = 166)	Resistance Group (n = 65)	Multivariate Analysis		Univariate Analysis	
				*F*/χ^2^	*P*	*t*/χ^2^	*P*
Mean age ± SDs, yr	7.7 ± 4.0	7.4 ± 3.7	8.5 ± 4.6	3.380	0.067	−1.838	0.067
Sex (M/F)							
Male	139 (60)	104 (63)	35 (54)	1.668	0.196	1.668	0.196
Female	92 (40)	62 (37)	30 (46)				
Body mass index, kg/m^2^	18.2 ± 4.0	17.9 ± 3.3	18.3 ± 4.2	0.685	0.409	−0.828	0.409
Type of seizure, n (%)							
Generalized onset	44 (19)	31 (19)	13 (20)	17.672	0.001*	0.032	0.858
Focal onset	90 (39)	75 (45)	15 (23)			10.784	0.001*
Combined generalized and focal onset	53 (23)	27 (16)	26 (40)			14.286	<0.001†
Unknown onset	44 (19)	33 (20)	11 (17)			0.298	0.585
LCM dose [mg/(kg·d)]	7.7 ± 2.4	7.8 ± 2.1	7.7 ± 2.8	0.000	0.987	0.015	0.987
LCM concentration, μg/mL	6.3 ± 2.9	6.3 ± 2.8	5.3 ± 2.4	5.558	0.019*	2.534	0.012*
LCM CD, μg/mL per kg/mg	0.9 ± 0.4	0.9 ± 0.4	0.7 ± 0.3	4.805	0.029*	2.192	0.029*
LCM treatment							
Monotherapy	52 (23)	47 (28)	5 (8)	30.731	<0.001†	13.550	<0.001†
2 ASMs	95 (41)	76 (46)	19 (29)			6.165	0.013*
3 ASMs	61 (26)	30 (18)	31 (48)			20.353	<0.001†
4 ASMs	23 (10)	13 (8)	10 (15)			2.407	0.121

All clinical data were collected at the last follow-up (at 3, 6, or 12 months after starting LCM therapy) of the pediatric patients. LCM serum concentrations were obtained during a patient's chronic, unchanged/uninterrupted LCM dosing (approximately 12 h after the evening dose, trough concentration).

* *P* value < 0.05.

† *P* value < 0.001.

The patients were divided into 2 groups: the LCM-responsive group (n = 166, 72%) and the LCM-resistant group (n = 65, 28%). Multivariate analysis of factors that affect LCM response revealed no significant differences in age, sex, body mass index, and LCM dose between the responsive and resistant groups (*P* > 0.05). However, we observed significant differences in the types of seizures, plasma LCM concentration, concentration-to-dose (CD) ratio, and concomitant ASMs between the responsive and resistant groups (*P* < 0.05) (Table 2).

To further analyze the effect of each factor on the response of patients to LCM, the data on each factor were compared between the drug-resistant and drug-responsive groups using the χ^2^ (χ2) test or Fisher exact test. Furthermore, the Student *t* test or the Mann–Whitney *U* test was conducted for analysis of quantitative variables. We observed significant differences in the type of seizure (focal onset and a combination of generalized and focal onset), plasma LCM concentrations, CD ratio, and use of monotherapy, 2 concomitant ASMs, or 3 concomitant ASMs between the drug-responsive and drug-resistant groups (*P* < 0.05; Table 2).

### Genotype and Allele Frequencies of *ABCC2*

All the *ABCC2* polymorphisms studied were found to follow the Hardy–Weinberg equilibrium in LCM-responsive and LCM-resistant patients (*P* > 0.05), indicating that the research subjects were representative of the entire group.

Interestingly, the proportion of patients with the *ABCC2* 1249G>A (rs2273697) AA genotype in the drug-resistant group was significantly higher than that in the drug-responsive group (*P* < 0.05; Table 3). In addition, the proportion of patients with the *ABCC2* −24C>T (rs717620) TT genotype in the drug-resistant group was significantly higher than that in the drug-responsive group (*P* < 0.05; Table 3). Furthermore, the proportion of patients with the *ABCC2* 1249G>A (rs2273697) A allele and *ABCC2* −24C>T (rs717620) T allele in the drug-resistant group was significantly higher than that in the drug-responsive group (*P* < 0.05; Table 3).

**TABLE 3.** Genotype, Haplotype, and Diplotype Frequencies of ABCC2 Polymorphisms in Pediatric Patients With Epilepsy in the Effective (n = 166) and Ineffective (n = 65) Groups

SNP	Genotype	Genotype Frequencies			Odds Ratio (95% Confidence Interval)	χ^2^	*P*
		Total Group, n (%)	Responsive Group (n = 166)	Resistance Group (n = 65)			
*ABCC2* 1249G>A (rs2273697)	GG	90 (39)	77 (46)	13 (20)	3.407 (1.818–6.387)	15.287	<0.001*
	GA	103 (45)	70 (42)	33 (51)	0.696 (0.398–1.216)	1.628	0.202
	AA	38 (16)	19 (12)	19 (29)	0.334 (0.159–0.701)	8.866	0.003†
	G#	283 (61)	224 (67)	59 (45)	2.481 (1.398–4.404)	9.821	0.002†
	A#	179 (39)	108 (33)	71 (55)			
*ABCC2* −24C>T (rs717620)	CC	101 (44)	84 (51)	17 (26)	2.962 (1.635–5.368)	13.198	<0.001*
	CT	95 (41)	62 (37)	33 (51)	0.564 (0.321–0.992)	3.977	0.046†
	TT	35 (15)	20 (12)	15 (23)	0.457 (0.213–0.978)	4.190	0.041†
	C^#^	297 (64)	230 (69)	67 (52)	2.055 (1.153–3.660)	6.047	0.014†
	T^#^	165 (36)	102 (31)	63 (48)			
Haplotype	G–C	174 (36)	134 (40)	40 (26)	1.897 (1.042–3.457)	4.432	0.035†
	G–T	94 (19)	64 (19)	30 (20)	0.938 (0.466–1.889)	0.032	0.858
	A–C	108 (22)	71 (21)	37 (25)	0.797 (0.412–1.544)	0.452	0.502
	A–T	111 (23)	67 (20)	44 (29)	0.612 (0.319–1.176)	2.189	0.139
Diplotype	GG–CC	71 (31)	62 (37)	9 (14)	3.608 (1.799–7.233)	13.923	<0.001*
	GG–CT	17 (7)	13 (8)	4 (6)	1.362 (0.455–4.080)	0.307	0.579
	GA–CC	28 (12)	21 (13)	7 (11)	1.209 (0.514–2.844)	0.189	0.663
	GA–CT	58 (25)	38 (23)	20 (31)	0.665 (0.354–1.248)	1.624	0.203
	GA–TT	17 (7)	11 (7)	6 (9)	0.761 (0.272–2.130)	0.272	0.602
	AA–CT	20 (9)	11 (7)	9 (14)	0.462 (0.178–1.200)	2.607	0.106
	AA–TT	16 (7)	7 (4)	9 (14)	0.256 (0.081–0.807)	6.105	0.013†
	Others‡	4 (2)	3 (2)	1 (1)	2.020 (0.180–22.645)	0.338	0.561

The drug-responsive group was compared with the drug-resistant group.

* *P* value < 0.001.

† *P* value < 0.05.

‡ Haplotype and diplotype with total frequencies below 5% in the 2 groups.

### Haplotype and Diplotype Frequencies

The frequencies of 2-marker haplotypes are presented in Table 3. We estimated 4 possible haplotypes, which were then compared between the LCM-responsive and LCM-resistant groups. All haplotypes were found to be overrepresented at a percentage higher than 10%. In addition, the G-C haplotype carrier frequency was significantly higher in the LCM-responsive group than in the LCM-resistant group (*P* < 0.05, OR = 1.897, 95% CI = 1.042–3.457).

We also conducted diplotype analysis of the 1249G>A and 24C>T polymorphisms. Our results suggested that the frequency of GG-CC diplotypes carriers was significantly higher in the LCM-responsive group than in the LCM-resistant group (*P* < 0.001; OR = 3.608, 95% CI = 1.799–7.233; Table 3). However, the AA-TT diplotypes carrier frequency was significantly lower in the LCM-responsive group than in the LCM-resistant group (*P* < 0.05, OR = 0.256, 95% CI = 0.081–0.807).

### Relationships Between Genetic Polymorphisms and Therapeutic Efficacy

To investigate the effects of genetic polymorphisms on LCM responsiveness, patients were subdivided into different groups according to the SNP. Both *ABCC2* 1249G>A (rs2273697) and *ABCC2* −24C>T (rs717620) exhibited a significant correlation with response to LCM treatment.

As presented in Table 3, the number of carriers of the *ABCC2* 1249G>A (rs2273697) AA genotype was higher among LCM-resistant patients than among LCM-responsive patients (*P* < 0.05; OR = 0.334, 95% CI = 0.159–0.701; Table 3). Moreover, the number of *ABCC2* 1249G>A (rs2273697) GG genotype carriers was higher among LCM-responsive patients than among LCM-resistant patients (*P* < 0.001; OR = 3.407, 95% CI = 1.818–6.387). Compared with patients carrying the A allele, more patients carrying the G allele showed good efficacy (*P* < 0.05, OR = 2.481, 95% CI = 1.398–4.404).

In addition, the number of patients carrying the *ABCC2* −24C>T (rs717620) CT and TT genotypes was higher in the LCM-resistant group than in the LCM-responsive group (*P* < 0.05; OR = 0.564, 95% CI = 0.321–0.992; *P* < 0.05; OR = 0.457, 95% CI = 0.213–0.978). We also discovered more patients carrying the *ABCC2* −24C>T (rs717620) CC genotype in the LCM-responsive group than in the LCM-resistant group (*P* < 0.001; OR = 2.962, 95% CI = 1.635–5.368). Compared with patients carrying the T allele, more patients carrying the C allele showed good efficacy (*P* < 0.05, OR = 2.055, 95% CI = 1.153–3.660).

### Associations Between *ABCC2* Polymorphisms and Plasma LCM Concentration

The mean LCM dose of the patients at the last follow-up (at 3, 6, or 12 months after the start of LCM therapy) was 7.8 ± 2.1 mg/(kg·d) and 7.7 ± 2.8 mg/(kg·d) in the LCM-responsive and LCM-resistant groups, respectively. In addition, the mean plasma LCM concentration in the LCM-responsive group (6.3 ± 2.8 mcg/mL) was significantly higher than that in the LCM-resistant group (5.3 ± 2.4 mcg/mL). The mean LCM CD ratio of the LCM-responsive group (0.9 ± 0.4 mcg/mL per kg/mg) was also significantly higher than that of the LCM-resistant group (0.7 ± 0.3 mcg/mL per kg/mg).

We discovered that the *ABCC2* 1249G>A and *ABCC2* −24C>T polymorphisms had a significant influence on plasma LCM concentration and LCM CD ratio (Table 4). Patients with the *ABCC2* 1249G>A GA genotype (5.6 ± 2.4 mcg/mL) and AA (4.1 ± 1.8 mcg/mL) genotype exhibited a significantly lower plasma LCM concentration than patients with the GG genotype (7.3 ± 2.8 mcg/mL) (*P* < 0.001; Table 4). Furthermore, patients with the *ABCC2* 1249G>A GA genotype (0.7 ± 0.3 mcg/mL per kg/mg) and AA genotype (0.5 ± 0.3 mcg/mL per kg/mg) had a significantly lower LCM CD ratio than patients with the GG genotype (1.0 ± 0.4 mcg/mL per kg/mg) (*P* < 0.001; Table 4).

**TABLE 4.** Effects of the ABCC2 Genotypes on Adjusted Plasma LCM Concentrations

SNP	Genotype	Number (%)	Plasma Concentration, μg/mL	*F*/ *t*	*P*	95% Confidence Interval	CD, μg/mL per mg/kg	*F*/ *t*	*P*	95% Confidence Interval
ABCC2 1249G>A (rs2273697)	GG	90 (39)	7.3 ± 2.8	—	—	—	1.0 ± 0.4*	—	—	—
	GA	103 (45)	5.6 ± 2.4	4.448	<0.001†	0.938–2.434	0.7 ± 0.3	6.328	<0.001†	0.219–0.418
	AA	38 (16)	4.1 ± 1.8	6.444	<0.001†	2.227–4.202	0.5 ± 0.3	7.794	<0.001†	0.373–0.628
	GG	90 (39)	7.3 ± 2.8	6.122	<0.001†	1.423–2.773	1.0 ± 0.4	7.864	<0.001†	0.275–0.460
	GA + AA	141 (61)	5.2 ± 2.3				0.7 ± 0.3			
ABCC2 −24C>T (rs717620)	CC	101 (44)	7.4 ± 3.0	—	—	—	1.1 ± 0.4‡	—	—	—
	CT	95 (41)	5.1 ± 1.9	6.459	<0.001†	1.608–3.022	0.6 ± 0.2	9.403	<0.001†	0.347–0.531
	TT	35 (15)	4.4 ± 2.0	5.597	<0.001†	1.944–4.069	0.6 ± 0.3	7.232	<0.001†	0.333–0.586
	CC	101 (44)	7.4 ± 3.0	7.727	<0.001†	1.863–3.139	1.1 ± 0.4	10.428	<0.001†	0.361–0.529
	CT + TT	130 (56)	4.9 ± 1.9				0.6 ± 0.2			

* The ABCC2 1249G>A (rs2273697) GG genotype compared with the GA genotype or AA genotype.

† *P* value < 0.001.

‡ The ABCC2 −24C>T (rs717620) CC genotype compared with the CT genotype or TT genotype.

Moreover, patients with the *ABCC2* −24C>T CT genotype (5.1 ± 1.9 mcg/mL) and TT genotype (4.4 ± 2.0 mcg/mL) exhibited a significantly lower plasma LCM concentration than patients with the CC genotype (7.4 ± 3.0 mcg/mL) (*P* < 0.001; Table 4). Furthermore, patients with the *ABCC2* −24C>T CT genotype (0.6 ± 0.2 mcg/mL) and TT genotype (0.6 ± 0.3 mcg/mL) showed a significantly lower LCM CD ratio than patients with the CC genotype (1.1 ± 0.4 mcg/mL per kg/mg) (*P* < 0.001; Table 4).

## DISCUSSION

LCM has an extensive interindividual pharmacokinetic variability, partly owing to genetic polymorphisms in drug transporters.^22^ Our study comprehensively evaluated the relationship between the *ABCC2* transporter and the plasma concentration and clinical efficacy of LCM in Uygur pediatric patients with epilepsy who received LCM treatment. The findings of this study indicate that the *ABCC2* 1249G>A (rs2273697) AA genotype and *ABCC2* −24C>T (rs717620) TT genotype are significantly correlated with lower plasma LCM concentrations and LCM CD ratios than the GG genotype and CC genotype (*P* < 0.001) in LCM-resistant patients. To the best of our knowledge, this is the first study to investigate the relationship between genetic polymorphisms in drug transporters and plasma LCM concentrations.

First, we analyzed the demographic characteristics of pediatric patients in the LCM-responsive and LCM-resistant groups. In the LCM-resistant group, the proportion of combined generalized and focal onset seizure was significantly higher than of other seizure types (*P* < 0.05). Furthermore, in the LCM-responsive group, the proportion of focal onset seizure was significantly higher than that of any other seizure type (*P* < 0.05). The reason may be the higher number of pediatric patients with combined generalized and focal onset seizure in the drug-resistant group. Perrenoud et al^23^ observed no differences in plasma LCM levels between responders (median 10.4 mcg/mL) and nonresponders (median 9.5 mcg·mL-1; *P* = 0.36), even after adjusting for additional predictors of clinical outcome. However, our study revealed that the mean plasma LCM concentration and LCM CD ratio in the LCM-resistant group were significantly lower than those in the LCM-responsive group (*P* < 0.05). The results of this study were different from those of Perrenoud et al.^23^

Previous studies have reported ABCB1, SCN1A, SCN2A, ATP1A2, and ATP1A3 as genetic factors associated with drug resistance in epileptic patients.^24–27^ However, only a few studies have evaluated the relationship between *ABCC2* polymorphisms and drug resistance in patients with epilepsy. Several studies have shown that the relationship between *ABCC2* polymorphisms and drug resistance is not consistent across different geographical regions and countries.^15–17,28,29^ Grewal et al^15^ revealed that alterations in the functionality of *ABCC2* can affect the disposition and bioavailability of drugs, which interferes with ASM therapy. A meta-analysis by Grover et al indirectly suggests a possible role of the *ABCC2* transporter at the blood–brain barrier in altering patient response to ASMs.^28^ Moreover, Ufer et al^29^ suggested a higher risk of ASM failure in *ABCC2* −24C>T allele carriers.

However, Seo et al,^28^ Kim et al,^29^ and Kim et al^30^ found no association between *ABCC2* polymorphisms and resistance to ASMs (including valproic acid, sultiame, phenobarbital, carbamazepine, oxcarbazepine, and lamotrigine) in young patients with epilepsy and adults with drug-refractory epilepsy.^28–30^

Our current results suggested that *ABCC2* 1249G>A (rs2273697) and *ABCC2* −24C>T (rs717620) were significantly related to LCM treatment response. The AA genotypes of *ABCC2* 1249G>A (rs2273697) were more frequent among patients who were LCM resistant. In addition, the number of carriers of *ABCC2* −24C>T (rs717620) CT and TT was higher among LCM-resistant patients than among LCM-responsive patients. Moreover, the number of carriers of the *ABCC2* 1249G>A (rs2273697) GG genotype and *ABCC2* −24C>T (rs717620) CC genotype was higher in the LCM-responsive group than in the LCM-resistant group (*P* < 0.05).

Our results are consistent with the findings of previous studies by Grewal et al,^15^ Grover et al,^28^ and Ufer et al.^29^ However, they were inconsistent with the results published by Seo et al,^30^ Kim et al,^31^ and Hilger et al.^32^ These discrepancies are likely attributed to ethnic differences in the frequencies of *ABCC2* genotypes and haplotypes. In addition, modern DNA studies have shown that the Uygur population has 2.6% Western Eurasian-specific haplogroups, whereas the Han population does not.^33^ Moreover, the Uyghur population is relatively isolated and has low mobility, strong genetic polymorphisms, and complex genetic structure. Therefore, there may be differences in the transport/metabolism pathways of ASMs between the Uygur and Han populations. These results emphasized the need for investigating the functional significance of *ABCC2* polymorphisms across different ethnic groups.

Several studies have indicated that *ABCC2* polymorphisms may be related to ASM concentrations and responsiveness to ASMs (such as valproic acid and oxcarbazepine).^34–36^ Chen et al discovered that valproic acid concentration in patients with the *ABCC2* rs2273697 AA genotype was significantly higher than that in patients with the GA + GG genotype (*P* = 0.000). This result suggests that *ABCC2* polymorphisms can affect valproic acid concentration and, consequently, treatment outcome in patients with epilepsy receiving valproic acid monotherapy.^34^ Yang et al reported that the *ABCC2* rs2273697 polymorphism was significantly related to oxcarbazepine plasma concentration in the whole patient cohort and in patients stratified by age (*P* = 0 0.033).^35^ However, Shen et al^36^ reported that polymorphisms of *ABCC2* rs2273697 were not associated with the concentrations and therapeutic efficacy of oxcarbazepine.

No reports are available on the relationship between *ABCC2* polymorphism with the plasma concentrations and efficacy of LCM in pediatric patients with epilepsy. This study is the first to evaluate the effect of *ABCC2* polymorphisms on the plasma concentrations and efficacy of LCM in Uygur pediatric patients with epilepsy. Our current study results show that *ABCC2* 1249G>A and *ABCC2* −24C>T (rs717620) polymorphisms have a significant effect on plasma LCM concentration. A significantly lower plasma LCM concentration was observed in *ABCC2* 1249G>A (rs2273697) AA genotype and *ABCC2* −24C>T (rs717620) TT genotype carriers, compared with that in GG genotype carriers (*P* < 0.001). Thus, the findings of this study suggest that the *ABCC2* 1249G>A (rs2273697) AA genotype and *ABCC2* −24C>T (rs717620) TT genotype can decrease P-gp activity, increase the gastrointestinal absorption of LCM, and ultimately decrease plasma LCM concentration.

Our study, however, had some limitations. First, pediatric patients present with limitations such as poor compliance to drug dosing. Second, considering that there are multiple factors that can affect LCM pharmacokinetics and treatment outcomes, there is always a possibility of confounders, including other SNPs. Third, regional differences, evaluation of clinical efficacy, judgment of epileptic drug resistance, and patient evaluation of their own condition also limited this study. Finally, the sample size was small. Therefore, the relationship between *ABCC2* polymorphisms and plasma LCM concentrations in patients with epileptic needs to be verified using ambidirectional methods and a larger sample size.

## CONCLUSIONS

The results of this study indicate that the *ABCC2* 1249G>A (rs2273697) and *ABCC2* −24C>T (rs717620) polymorphisms affect the plasma concentrations and treatment efficacy of LCM in pediatric patients with epilepsy, leading to drug resistance. In clinical practice, *ABCC2* polymorphisms should be identified before the start of LCM therapy. Plasma LCM concentrations should be monitored, and the dose should be readjusted for pediatric patients with epilepsy accordingly.
